# Living with age-related hearing loss and insights for hearing care: a qualitative descriptive study

**DOI:** 10.1080/17482631.2026.2652074

**Published:** 2026-03-30

**Authors:** Dukyoo Jung, Soogyung Shin, Sukyung Byeon, Leeho Yoo, Hyein Seo

**Affiliations:** a College of Nursing, Ewha Womans University, Seoul, South Korea

**Keywords:** Hearing impairment, presbycusis, aged, qualitative research, hearing aids

## Abstract

**Objectives:**

Age-related hearing loss is a common chronic condition that affects nearly one-fifth of the global population. It severely impairs communication, leading to social isolation, depression, and reduced quality of life of older adults. Understanding the difficulties and lived experiences of older adults is critical for improving hearing care strategies and communication outcomes.

**Design:**

A descriptive phenomenological qualitative design was employed to describe the experiences of older adults with age-related hearing loss from their own perspectives. Twelve participants aged ≥ 65 years with hearing loss were recruited using purposive sampling. Data were collected through semi-structured, face-to-face interviews.

**Results:**

Qualitative analysis identified four main themes, nine subthemes, and twenty-five meaningful statements. The themes reflected the lived experience of age-related hearing loss: 1) loss of “normalcy”, 2) early-stage internal struggles, 3) barriers to hearing aid use, and 4) efforts to live alongside hearing loss.

**Conclusions:**

Age-related hearing loss extends beyond auditory decline and includes psychological, social, and relational challenges. Addressing these challenges through education, group-based approaches involving peers and communication partners, and therapeutic communication is essential to improve hearing aid adherence, promote adaptive coping strategies, and support acceptance of hearing loss. Our findings provide a strong foundation for developing comprehensive interventions and long-term support for older adults with age-related hearing loss.

## 1. Introduction

Age-related hearing loss (ARHL), or presbycusis, is defined as a reduced ability to perceive sound and is commonly operationalized as a pure-tone audiometry threshold greater than 25 dB (Health Insurance Review & Assessment Service (HIRA), 2024; World Health Organisation (WHO), 2021). Globally, approximately 20% of the population experiences hearing loss, and this number is projected to rise from approximately 1.58 billion in 2019 to 2.49 billion by 2050 (Lee et al., 2025; World Health Organisation (WHO), 2021). ARHL is a major public health concern that profoundly affects people’s daily lives by disrupting communication, limiting social interactions, and undermining emotional well-being (Parmar et al., 2022).

For older adults, hearing loss profoundly disrupts everyday communication and social engagement. Difficulties in understanding speech, particularly in noisy environments or group conversations, limit participation in family interactions, community activities, and public life (Wasano et al., 2024). These persistent difficulties contribute to social isolation, loneliness, depression, and ultimately, diminished quality of life (Olsson et al., 2021; Tavanai et al., 2023). Daily communication is further complicated for older adults with hearing loss by external factors such as background noise, acoustic conditions, and the degree to which communication partners are aware of and adapt to their needs (Jain et al., 2019). These challenges reduce opportunities for social participation and foster feelings of marginalisation, underscoring the need to understand these individuals’ lived experiences to inform effective support strategies (Aldè et al., 2025). Yet evidence remains limited on how they sustain social participation and negotiate relationships in such in group-based community settings (Mamo et al., 2022). Understanding how older adults perceive and navigate these communication difficulties can guide the development of effective interventions, personalised hearing rehabilitation, and comprehensive care approaches to address their broader needs (Mamo et al., 2022).

In addition, ARHL is closely linked to cognitive vulnerability, and hearing rehabilitation has been discussed as a potential protective factor; however, despite these implications, hearing aid uptake among older adults remains low, and the contextual reasons for nonuse or inconsistent use—shaped by everyday routines, expectations, stigma-related concerns, and interpersonal dynamics—are not sufficiently elucidated (Lin et al., 2023; Mamo et al., 2022). Furthermore, ARHL shows robust associations with mental health outcomes such as depression (Lawrence et al., 2020), but targeted research is still limited in clarifying how emotional changes unfold within the lived context of recurrent communication failures, social withdrawal, and efforts to maintain normalcy in later life.

Despite the importance of understanding older adults’ experiences with hearing loss, most related research has relied on quantitative methods such as audiometric measures and standardised assessments (Neal et al., 2022), which are limited in capturing how communication difficulties are experienced and managed within older adults’ everyday living contexts, cognitive vulnerability, and social needs. Phenomenological research offers a powerful lens for exploring meaning and personal accounts of communication, allowing researchers to examine contextual and emotional dimensions that conventional measures cannot address (Coco et al., 2019).

Therefore, to address these gaps, this study explored the lived communication experiences of older adults with hearing loss. Using a phenomenological approach, it focused on the qualitative aspects of everyday challenges and the strategies older adults employ to manage communication barriers. This study can contribute to the development of effective nursing interventions for older adults with ARHL by offering in-depth insights into their perceptions of the condition, emotional barriers, and adaptation processes across diverse contexts.

## 2. Materials and methods

### 2.1. Study design

This study aimed to explore and understand the lived experiences of older adults with ARHL, focusing on their communication difficulties, hearing aid usages, and coping strategies. This study followed the Consolidated Criteria for Reporting Qualitative Research (COREQ) guidelines (Tong et al., 2007) (Supplemental Digital Content 1).

This study employed a descriptive phenomenological research design appropriate for revealing the essential and universal structures of the phenomenon. This design has a high degree of freedom in describing a phenomenon, thus allowing participants to describe their experiences and views from their own perspectives (Holloway & Galvin, 2023). Therefore, this method is appropriate for revealing the essence of the experiences of older adults with ARHL.

### 2.2. Participants

Purposive sampling was used to recruit older adults with ARHL because it can provide rich data from in-depth interviews (Holloway & Galvin, 2023). A gerontological nurse practitioner from a senior welfare centre in Seoul, South Korea, identified and evaluated the participants. The nurse practitioner referred eligible participants to a member of the research team based on the inclusion and exclusion criteria provided by the team. The inclusion criteria were as follows: (1) 65 years of age or older, (2) diagnosed with ARHL or presbycusis, and (3) able to express thoughts and feelings without assistance. Individuals who were unable to communicate because of cognitive-related diseases were excluded. Those who agreed to participate were included after receiving an explanation of the study design and purpose. Twelve older adults consented to participate in the study, with no dropouts.

### 2.3. Data collection

Data were collected through semi-structured interviews conducted in May 2025 that allowed participants to share rich and in-depth descriptions of their daily lives and experiences (Holloway & Galvin, 2023). One-on-one, face-to-face interviews were held in a quiet private programme room at a senior welfare centre, scheduled at the most convenient time for each participant.

The interview guide was initially developed by a research team member (SGS) and was grounded in the objectives of the study and existing literature. It was subsequently reviewed by a doctoral student (SKB) with qualitative research experience and a nursing professor specialising in gerontological research. The interviews were structured into four phases: introduction, transition, key questions, and closing. The key questions focused on communication difficulties, emotional responses, coping strategies, and experiences with hearing aid use (Table I).

**Table I.** Interview outline.

1. Introduction How are you spending your days? 2. Transition When was the first moment you noticed that your hearing was not the same as before, that you could not hear well? 3. Key questions What is the most inconvenient or difficult experience you face in daily life due to hearing difficulties? Have you ever had to ask others to repeat themselves or experienced misunderstandings because of your hearing loss? How has your hearing loss affected your relationships with people around you? How has your hearing loss affected your emotions? What methods of your own have you tried to communicate more effectively? What benefits and inconveniences have you experienced from using a hearing aid? 4. Closing In hindsight, how do you feel your hearing loss has affected your life?

All interviews were conducted in Korean by two female researchers (SGS and SKB), both of whom had professional clinical nursing experiences with caring older adults. Each interview lasted between 40 and 70 minutes. Field notes were taken throughout the interviews to document nonverbal cues, such as participants’ facial expressions, gestures, and posture changes. These observations were used to provide contextual support for interpreting verbal data, rather than being analysed as independent analytic data. The interview environment was adapted as needed by adjusting the seating proximity or speaking volume to ensure the participants’ comfort. Each interview was audio-recorded and transcribed verbatim by the researchers. The participants were asked directly to clarify any unclear words or phrases to ensure transcription accuracy.

Following data collection, analysis, and manuscript preparation were conducted in Korean. After the manuscript was completed, an initial English draft was prepared by a research team member (SGS), who is fluent in both Korean and English, and was subsequently reviewed by a professional translator with experience in academic manuscript translation. The reviewed English manuscript was then back-translated into Korean, and the research team members compared it with the original version to verify consistency in terminology, expressions, and meaning.

Across the statements of the final 12 participants, similar content and meanings were repeatedly observed, and no new meaningful statements were identified. Accordingly, the research team determined that sufficient data had been collected regarding experiences of living with hearing loss and hearing care insights, and this point was considered to represent data saturation. Therefore, data collection was concluded.

### 2.4. Data analysis

The collected data were tagged on meaningful segments and organised and stored by themes using Taguette (version 1.5.2; Rémi Rampin, New York University, New York, NY, USA), a free open-source qualitative data management tool, after which the research team conducted the analysis.

We adopted Colaizzi’s phenomenological method as an analytical framework, which enabled a systematic and in-depth examination of the data (Colaizzi, 1978). The process included the following steps. (1) The authors (SGS, SKB, and LHY) read the transcriptions repeatedly to become immersed in the data. All researchers who conducted the interviews were included. (2) Significant statements related to the experiences of older adults with ARHL were identified. (3) Three authors (SGS, SKB, and LHY) derived formulated meanings from the statements in the first round. In the second round, the extracted meanings were cross-checked. (4) The formulated meanings were organised into subthemes and themes through regular team discussions. Any conflicts among the researchers were addressed during these meetings. (5) The themes were linked closely to the research phenomenon and described in detail. (6) The data were repeatedly grouped and reorganised to avoid repetition and describe the phenomenon concisely until all authors reached a consensus. (7) The results were presented to one participant to ensure authenticity and validate the essence of the experience.

### 2.5. Rigour

We applied Guba and Lincoln’s (Guba & Lincoln, 1994; Sharifzadeh, 2024) criteria to ensure the rigour of this qualitative study. For credibility, all authors involved in the analysis had completed graduate-level coursework in qualitative research and possessed substantial expertise in gerontological studies. The findings were shared with one participant to verify the accuracy of the researchers’ interpretations. We provided detailed descriptions of the study design, sampling procedures, inclusion and exclusion criteria, and data collection methods before the interviews to support transferability. The same researchers who conducted the interviews also participated in the data analysis to ensure dependability. Furthermore, themes and subthemes were developed through consensus during regular team meetings. Finally, we ensured confirmability by engaging in reflexive discussions regarding our preconceptions and potential biases. We employed bracketing to minimise their influence. Because all authors were professional nurses with hands-on clinical experience in older adult care, we continually reflected how our professional identities might influence both the interview process and data interpretation, particularly by increasing our sensitivity to healthcare-related concerns throughout data collection and analysis. During the interviews, the participants were provided sufficient time to respond without interruption, allowing for richer descriptions of their experiences.

### 2.6. Ethical considerations

This study was approved by the Ewha Womans University Institutional Review Board (IRB No. ewha-202502-0011-02). All participants were fully informed about the purpose of the study and procedures and were assured that they could withdraw at any time for any reason without any impact on their care. Written informed consent was obtained from all participants after they were provided with sufficient information about the study. Each participant was assigned an anonymous code before data analysis to maintain confidentiality.

## 3. Results

Table II presents the general characteristics of the participants. The participants were 12 older adults with hearing loss, with an equal number of male and female participants. The mean age was 81.67 years (SD = 3.3; range, 74–87), and three participants lived alone. None of the participants were employed. The mean estimated duration since the diagnosis of ARHL was 7.17 years (SD = 2.76; range, 2–10). Eight participants reported “frequent” hearing aid use (≥15 days per month), two reported “occasional” use (<15 days per month), and two reported no use (“none”).

**Table II.** Participant characteristics (*N* = 12).

Participants	Age (years)	Sex	Living alone	Current working	Estimated years since ARHL diagnosis, (years)	Hearing aid use
1	82	Female	No	No	7	None
2	83	Female	No	No	3	Frequent
3	82	Male	No	No	5	None
4	82	Male	Yes	No	10	Frequent
5	82	Female	No	No	8	Frequent
6	74	Female	Yes	No	6	Frequent
7	87	Female	No	No	2	Occasional
8	83	Male	No	No	7	Frequent
9	81	Male	No	No	8	Occasional
10	82	Male	No	No	10	Frequent
11	80	Female	Yes	No	10	Frequent
12	76	Male	No	No	10	Frequent

ARHL: Age-related hearing loss.

Hearing aid use: Frequent (15 days or more per month), Occasional (less than 15 days per month).

The qualitative analysis identified four main themes, nine subthemes, and twenty-five meaningful statements describing the communication experiences and daily lives of older adults with ARHL (Table III).

**Table III.** Experiences of older adults with ARHL.

Theme	Subtheme	Meaningful Statement
Loss of “normalcy”	Functional difficulties in daily life	Daily life became inconvenient due to impaired hearing
		Experienced losses due to mishearing
	Impaired emotional well-being	Anxiety stemming from missed auditory warning signals
		Discomfort caused by amplified everyday sounds
		Reduced vitality in life
	Misunderstandings and strain in relationships	Conflicts arising from communication breakdowns
		Feeling marginalised and disconnected
Early-stage internal struggles	Inner conflict in the process of acceptance	Resigned acceptance of the inevitability
		Coexistence of denial and confusion
		Seeking personal explanations for hearing loss
	Withdraw and avoid communication	Concealing hearing difficulties
		Distancing oneself from conversations Conversations
		Gradually reducing participation in social activities
Barriers to hearing aid use	Hearing aids as limited but necessary	Difficulties in using hearing aids
		Hearing aids not meeting expectations
		Acknowledging hearing aids as essential for daily life
	Insufficient support and financial burden	Financial burden of purchasing hearing aids
		Desire for government-provided support
Efforts to live alongside hearing loss	Active coping strategies	Disclosing hearing difficulties
		Adjusting communication environments
		Utilising alternative communication methods
		Using residual senses to support communication
	Efforts to maintain a positive outlook	Making consistent efforts to adapt to hearing aid use
		Encountering positive moments amid hearing loss
		Striving to maintain a positive self-image

## 4. Theme 1. Loss of “normalcy”

Participants experienced a sense of loss in “normalcy” as their hearing function declined, accompanied by changes across multiple areas of life. They described a disruption in everyday communication and self-identity as hearing deterioration affected their relationships, social participations, and autonomy. Practical difficulties in daily routines were accompanied by emotional distress, communication errors often led to interpersonal problems, and they gradually withdrew from social interactions, resulting in progressive social isolation. Three subthemes were identified: *cracks in daily life*, *misunderstandings and strain in relationships*, and *social withdrawal and isolation.*

### 4.1. Subtheme 1.1. Functional difficulties in daily life

Participants described various inconveniences and functional difficulties they had not experienced previously due to hearing loss. Reduced listening ability led to missing important information or difficulty understanding conversational contexts, resulting in challenges in communicating with others, thereby restricting basic activities such as administrative tasks and causing practical inconveniences and difficulties.

*“On some subway trains, the announcements are loud, so I can hear them. But on some trains, the announcements are quiet, so I can’t hear them. Then I end up going one stop too far or getting off one stop early.”* (P 6)

*“When I go to the hospital, doctors speak so quietly that I cannot understand what they are saying. I cannot keep asking them to repeat, so I just say, ‘Yes, yes,’ but often, I do not really know what I was told.”* (P 8)

*“I need to renew my driver’s license, but I haven’t done it yet because I feel hesitant to go there since I can’t hear well.”* (P 4)

### 4.2. Subtheme 1.2 Impaired emotional well-being

Hearing decline led participants to experience emotional difficulties beyond everyday inconvenience. Some felt anxious when auditory warning signals could not be heard and perceived their safety as threatened, while everyday noises were amplified, causing unpleasant sensations.

*Sometimes when a car honks behind me, I cannot hear it and fail to move out of the* *way.”* (P 2)

*“It makes me anxious because I can’t hear well. It’s not just one or two things. I live alone, so if there’s a fire at night, I wouldn’t hear the fire alarm, right?”* (P 4)

*The most uncomfortable thing is loud noises, like the sound of trucks passing by or* *cars on the street. I hate those. They sound much louder to me.”* (P 9)

These emotional distresses were closely interconnected and led not only to communication difficulties but also to diminished motivation for life and depressive symptoms among older adults.

*“I get very depressed, because communication doesn’t work. And it’s not like I can always write things down to communicate. So this feels really hard.”* (P 5)

*“I felt like I had become completely stupid, just because I couldn’t understand what* *others were saying. So, for a while, I stopped going out and just stayed home. But* *being at home made me feel depressed.”* (P 10)

### 4.3. Subtheme 1.3. Misunderstandings and strain in relationships

Hearing loss also damaged participants’ interpersonal relationships, often between family members and peers. Mishearing what others said caused delayed responses or distorted conversations that escalated into unnecessary conflicts. Changes in voice volume or tone were sometimes misinterpreted as anger. In particular, when both spouses had hearing difficulties, the strain was magnified, damaging the relationship.

*“Wearing hearing aids helps, but at home I often do not wear them. Then I fail to understand properly, give vague answers, and end up arguing.”* (P 8)

*“A few years ago, my wife’s hearing also declined, and things became even harder. Since neither of us could hear well, we had to speak loudly. But raising our voices made it look like we were always fighting or angry. That was distressing.”* (P 10)

Participants also felt alienated or excluded when others withdrew from deeper conversations after they revealed their hearing difficulties. This was particularly evident in interactions with acquaintances or friends and group conversations. One participant expressed negative feelings toward using a hearing aid because physicians appeared inattentive and ignored them at the hospital.

*“During conversations, people keep talking to me, but when I say I cannot hear well, they suddenly cut off the conversation. [….] It feels like they do not want to treat me as a conversation partner.”* (P 1)

*“When I talk one-on-one it’s okay, but when there are several people here, this person says something, and that person says something, I can’t hear well.”* (P 2)

*“Because I can’t hear, when people start laughing, I can’t laugh along.”* (P 5)

*“Doctors don’t really listen. They just recommend hearing aids as if it is a routine duty, without really paying attention.”* (P 1)

## 5. Theme 2. Early-stage internal struggles

Participants continuously experienced internal struggles during the early stages of hearing loss, after they recognised their hearing impairment. These struggles were closely associated with their perceptions of age-related hearing impairment and self-stigmatisation as “an elderly person who cannot hear.” Two subthemes were identified: *inner conflict in the process of acceptance* and withdraw *and avoid communication.*

### 5.1. Subtheme 2.1. Inner conflict in the process of acceptance

After becoming aware of their hearing loss, participants experienced various psychological conflicts during the acceptance process. As they realised their situation, feelings of loss and denial became pronounced, and they experienced psychological distress and depression while perceiving hearing loss not as merely a medical sensory impairment but as part of the aging process. In addition, participants attempted to understand their situation by linking their hearing loss to life experiences accumulated over the years and searching for possible causes of their hearing decline.

*“It is already hard enough getting old, but why do even things like this have to break down? I think my hearing worsened because I lived under so much stress. I endured 50 years of hardship in my marriage, and I believe that constant stress damaged me. That is my own thought…”* (P 2)

*“I always listened to music with earphones in the past and even worked with them in. That must have broken my ears.”* (P 1)

*“Because communication does not go smoothly, it is mentally exhausting. I have to keep asking questions, and that constant effort brings overwhelming psychological pain.” (P 4)*

*“When conversations don’t go well, I get very depressed… so I started learning singing. I love music, and it really helps me.”* (P 5)

Eventually, participants perceived their hearing decline as a natural part of aging and showed an attitude of acceptance. They did not try to resist or avoid their hearing loss but persuaded themselves that it comes naturally with the passage of time, adapting to reality within the aging process and accepting it with resignation.

*“When you get older, there is only one path to follow. I try to think, ‘This must be because of aging,’ and accept everything as it is. On the subway, when I say something, some people reply, ‘I can’t hear, I can’t hear.’ I remind myself that I should not compare myself to others who are better off.”* (P 9)

*“Since hearing loss has already come to me, I do not try to avoid it but live with it. If you resist it, you are only rushing your own fate. Time will pass anyway, so the question is how comfortably we can live with it.”* (P 12)

*“I think there is nothing I can do but accept it naturally. I tell myself, ‘This must be because I am aging,’ and try to live with it.”* (P 9)

### 5.2. Subtheme 2.2. Withdraw and avoid communication

Although participants struggled with communication difficulties, they often hesitated to disclose their hearing problems because of shame and concern about inconveniencing others. They often attempted to conceal their hearing problems by pretending to understand or passively engaging in conversations. Such attitudes eventually led them to withdraw from conversations or avoid group situations completely, thereby decreasing their participation in social activities.

*“When several people gather and talk, I can’t hear well, so I just don’t talk. I just sit quietly next to them. Since I can’t hear well, I don’t even try hard to catch it anymore. I just sit there looking at my phone.”* (P 3)

*“When there are many people, I can’t really understand what’s being said, so I want to avoid those situations. Even if I go to a gathering, it’s not enjoyable.”* (P 1)

*“I just don’t say that I can’t hear. Whatever they say, I just pretend I heard… It’s hard to admit that I can’t hear, so I just pretend I did and say ‘yes, yes.’”* (P 1)

*“When ordering something, if I pretend to understand without really hearing, I might end up paying more. That’s just how I live.”* (P 1)

*“With strangers, I rarely say that I can’t hear… When you meet someone for the first time, they don’t really understand. It feels uncomfortable and makes it hard to talk.”* (P 8)

*“Because I can’t hear well, I don’t go to reunion events. Even if I do go, I just eat and then make an excuse that I’m busy and leave. This time, too, I skipped the gathering.”* (P 11)

## 6. Theme 3. Barriers to hearing aid use

Participants repeatedly encountered barriers to hearing aid use and experienced ambivalent feelings of reliance and frustration. While they expressed dissatisfaction with the factors hindering consistent hearing aid use, they also acknowledged the indispensability of the devices. Two subthemes were identified: *hearing aids as limited but necessary* and *insufficient support and financial burden.*

### 6.1. Subtheme 3.1. Hearing aids as limited but necessary

Participants recognised hearing aids as necessary tools for communication and daily living. However, they reported inconveniences in using them, such as charging, itching, malfunctioning, and losing. They expressed frustration that their hearing aids did not meet their expectations, with sounds often echoing and words remaining unclear. Some participants even stopped using them. Nevertheless, they accepted hearing aids as unavoidable if they were to maintain social relationships and daily living. Even participants who did not wear them regularly carried the device and spare batteries “just in case.”

*“I always have to worry about charging or batteries. Once I forgot where I put it after taking it out, and it was such a hassle.”* (P 9)

*“Since it’s an electronic device, water causes damage. When I wash my face, I constantly worry that splashing water will break it.”* (P 8)

*“I thought hearing aids would let me hear well, but words actually sound less clear. For example, when someone says ‘full name,’ I hear it as something like ‘hul-ham’ Sounds are louder with this [hearing aid], but words are not distinct.”* (P 2)

*“How can I go out if I cannot understand conversations? With this device, at least I can manage one-on-one conversations even if group discussions are difficult. Now, without a hearing aid, I cannot live—I cannot even go out.”* (P 1)

*“Just like people rarely leave home without their phone, without hearing aids, communication even at home becomes impossible.”* (P 12)

*“I don’t wear hearing aids because they don’t help much… but I always carry them with batteries as a backup in case I might need them.”* (P 11)

### 6.2. Subtheme 3.2. Insufficient support and financial burden

Older adults typically do not have stable incomes, which can make hearing aid use financially burdensome. Continuous expenses for consumables, repairs, and maintenance added to the difficulty, leading some to delay or even give up replacing their devices. Furthermore, strict eligibility criteria for hearing aid insurance benefits made it difficult to access national support, and subsidies were often limited to initial purchase costs, rendering additional expenses, such as examination fees, unaffordable.

*“My hearing aid doesn’t work well now, so I should replace it, but it is difficult to afford. It’s a large expense, and since I have no income, I keep worrying about what to do.”* (P 2)

*“Hearing aids cost a lot and often break. Just fixing the microphone costs about 100,000 won. Batteries used to be 20,000 won for 40, but now it’s over 30,000. Even the cost of batteries can’t be ignored.”* (P 8)

*“It would be better if the government provided certified hearing aids. If high-quality devices were supported by the state, the burden would be lighter.”* (P 2)

*“After surgery, my hearing worsened, so I wanted hearing aids. But at first, I couldn’t get them because I wasn’t eligible for disability benefits. Later, I reapplied and was able to purchase them with government support.”* (P 10)

## 7. Theme 4. Efforts to live alongside hearing loss

Participants employed various coping strategies to minimise communication breakdowns. Through repeated difficult experiences, they acquired their own knowledge for smoother communication and sought to maintain a positive mindset while living with hearing loss. Two subthemes were identified: *active coping strategies* and *efforts to maintain a positive outlook.*

### 7.1. Subtheme 4.1. Active coping strategies

Participants actively adjusted their behaviour and environment despite communication restrictions. Most disclosed their hearing difficulties upfront and asked for understanding, which often prompted others to speak slowly or repeat themselves. One participant explained how repeated discomfort gave her the courage to speak first, stating, *“I started to have the courage because I was experiencing so many inconveniences when dealing with people” (P 6)*. They also chose quiet places for easier communication, used pen and paper, observed others’ lip movements, and attempted to learn sign language as alternative methods of interaction. In addition, they used and preferred text messages instead of phone calls, relied on captions, and utilised residual hearing to facilitate communication.

*“I speak first, otherwise people will not know. I let them know in advance—whether at the bank, the public health centre, or the bakery. That way I can get help. I always explain first.”* (P 2)

*“Since listening is the key to conversation, I guide people to meet in quiet places like a park when we need to talk. That is the wisdom of life.”* (P 12)

*“When it is really important, I go closer and ask them to write it down on paper. That way I can be sure, otherwise I might make a mistake.”* (P 5)

*“I perk up my ears and focus intently. My right ear is better, so I ask people to sit on my right side during conversations. That way, I can hear better.”* (P 7)

### 7.2 Subtheme 4.2. Efforts to maintain a positive outlook

Participants sought to reframe their perspectives and think positively to live with hearing loss. Despite the inconvenience of visiting hearing aid centres and discomfort of wearing the devices, participants tried to use them consistently until they became accustomed to them. Some even perceived their inability to hear as a positive aspect in certain situations. Furthermore, they engaged in hobbies to add vitality to their daily lives and strove to maintain a positive self-image by not inconveniencing others.

*“Since I do not hear well, if people speak louder, I can hear better, and that makes me feel good. More than anything, I feel grateful that they understand me and raise their voices for me.”* (P 2)

*“In the past, I sometimes went without wearing hearing aids, but now I think I must wear them consistently. I am trying to make it a habit, like second nature.”* (P 7)

*“Whenever people gossip, they whisper and point. If I had sharp hearing, I would catch it. But if I don’t hear it, then it doesn’t matter. It is better not to know.”* (P 6)

## 8. Discussion

The study aimed to understand the lived experience of older adults with ARHL. Twelve older adults were recruited from a senior centre in South Korea. The participants shared rich experiences about their daily challenges due to limited hearing ability, psychological distress in the early stages of hearing loss, hearing aid use, and strategies to adapt to the condition. The findings revealed four overarching themes: *loss of normalcy, early-stage internal struggles, barriers to hearing aid use, and continuous efforts to live alongside hearing loss*. These findings highlight that ARHL extends beyond auditory impairment to encompass identity, relationships, and overall quality of life.

### 8.1. Loss of “normalcy”

ARHL adversely affects multiple dimensions of older adults’ lives, often leading them to feel a sense of loss in their “normal life.” These findings are consistent with those of previous studies (Adachi & Paul, 2024; Holman et al., 2022), highlighting that older adults with hearing loss experience increased vulnerability in both functional and psychological aspects.

Most of the participants experienced impairments in everyday functioning due to their decreased ability to hear. This limitation altered their perception of daily life to be uncomfortable and threatening, leading them to view themselves as different from older adults with normal hearing. In later life, changes in perceived functioning can be particularly significant, as functional ability is a major component of healthy aging (World Health Organisation, 2020). Using assistive devices such as a cane, glasses, and hearing aids is commonly suggested to facilitate older adults’ participation in daily life (Lundin et al., 2022; St-Amour et al., 2019) and to enhance the sense of security (Lundin et al., 2022. However, most participants in this study still reported limitations in everyday functioning despite frequent hearing aid use. Within this context of functional vulnerability, hearing aids emerged in participants’ narratives as an expected means of support, yet one that did not fully restore their everyday functional difficulty. Therefore, future research is needed to examine how hearing aid use can be better aligned with everyday communication contexts to support the restoration of normalcy in older adults’ daily lives and to maintain functional ability.

Impairments in everyday functioning also heightened older adults’ psychological vulnerability, particularly in social interactions and communication with significant others. Older adults with ARHL frequently experience communication breakdowns and are often blamed for this by their conversation partners (Holman et al., 2022). These interactions trigger strong negative emotions, such as resentment and anger, in both parties, resulting in damaged relationships and decreased self-worth (Tseng et al., 2025). Furthermore, negative interpersonal experiences contribute to poor adherence to hearing aid use, reduced engagement in social activities, and difficulty accepting ARHL (Barker & Leighton, 2017; Davis et al., 2020). Barker and Leighton (2017) suggested that aligned coping strategies between older adults with hearing loss and their communication partners can support everyday social functioning. These findings indicate that education focused on aligned coping strategies with communication partners, such as spouses and family caregivers, may be essential for maintaining everyday emotional well-being in later life.

### 8.2. Early-stage internal struggles

In the early phase of ARHL, participants often avoided disclosing their hearing difficulties, pretended to understand conversations, and withdrew from social situations to avoid embarrassment or inconveniencing others. This was often accompanied by feelings of distress related to aging, as many participants expressed that “it is already hard enough getting old.” These findings suggest that older adults’ internal struggles with ARHL were reflected in frustration, concealment, withdrawal, and self-blame in everyday communication, indicating processes of internalised stigma. Previous studies have demonstrated that stigma related to hearing loss makes them give up, fosters withdrawal, and hinders the development of positive coping strategies (Barker & Leighton, 2017; Marcotti et al., 2024). Our findings extend this literature by illustrating how self-stigmatisation in older adults with ARHL is embedded in everyday communication practices and internal coping processes. According to Marcotti et al. (2024), peer group interventions can help normalise self-stigma, reduce feelings of isolation, and increase willingness to participate in social activities. For instance, the ACE (Active Communication Education) programme - a group-based intervention with peers—demonstrated improvements in self-reported communication outcomes and well-being (Hickson et al., 2007). Building on the literature, our findings suggest that interventions for older adults with ARHL may benefit from incorporating peer-based group settings that emphasise shared experiences while minimising the burden of disclosure.

### 8.3. Barriers to hearing aid use

The older adults with ARHL in this study expressed a love/hate relationship with hearing aids, which is consistent with previous studies (Holman et al., 2022; Pornprasit et al., 2024). Participants criticised the functional deficiencies, discomfort, and financial burden associated with hearing aids. However, most emphasised that hearing aids were essential for daily life and even for basic communication. Hearing aids play an important role in alleviating hearing-related symptoms and daily fatigue in older adults, increasing positive feelings and supporting independent functioning (Holman et al., 2022; Lundin et al, 2022; Tseng et al., 2025). This ambivalence highlights how hearing aid use is shaped by both perceived necessity and persistent dissatisfaction.

Healthcare providers’ communication style and interpersonal attitude significantly influence older adults’ engagement with rehabilitation services, hearing aid adoption, and continued use (Lundin et al., 2022). This was illustrated in our study, with one participant (P1) declining hearing aids because of negative communication experience with healthcare providers, underscoring their critical role in hearing aid adoption and acceptance. Therefore, these findings point to the importance of therapeutic communication by hearing healthcare providers that incorporates empathy, encouragement, and collaborative problem-solving in supporting sustained hearing aid use in daily life.

In addition, older adults’ ambivalence could be managed by increasing user satisfaction, which requires tailored device fitting, regular follow-ups, and training to hearing aid users (Solheim et al., 2017). Supporting older adults by combining auditory training that includes listening in noise with regular follow-up care may help increase satisfaction by promoting a successful communication experience (Van Wilderode et al., 2023). Furthermore, an active communication programme that incorporates peer discussions about communication strategies has been demonstrated to enhance social relationships and quality of life among older individuals who use hearing aids (Marcotti et al., 2024).

### 8.4. Efforts to live alongside hearing loss

Older adults with ARHL continuously made efforts to coexist with their condition by utilising diverse coping strategies. The strategies shown in our study, such as disclosing their hearing loss to others, adjusting their environment, and finding positive moments, align with the positive coping strategies found in previous studies (Tseng et al., 2025; Yuan et al., 2021). However, many of these coping strategies stemmed from the tendency to maintain a positive self-image by ‘not inconveniencing others’, which was also present in the early stages of ARHL. This socially oriented coping attitude has received limited attention in prior literature, underscoring the need for qualitative research to better understand how older adults interpret and manage their chronic condition. Given that hearing loss substantially affects self-identity (Holman et al., 2022), this tendency may serve as a strategy to preserve a sense of independence and competence in later life. Furthermore, the presence of this attitude from the early stages of ARHL aligns with prior literature suggesting that early coping patterns influence subsequent adjustments (Barker & Leighton, 2017).

While such coping strategies may help minimise social conflict and maintain relationships, they require continuous personal effort and may exacerbate emotional burden in the long term. Furthermore, coping strategies mentioned by participants were also largely managed at the individual level, which may limit help-seeking behaviour and may not be sustainable over time. Therefore, further research should be focus on developing organisational level interventions, such as group-based approaches in the community (Marcotti et al., 2024; Tseng et al., 2025), structured follow-up (Lundin et al., 2022), and multidisciplinary care models that coordinate audiologists, nurses, and otolaryngologists (Van Wilderode et al., 2023), to support older adults beyond individual-level efforts.

### 8.5. Strengths and limitations

This study underscores the value of qualitative data and a phenomenological approach in understanding the lived experiences of older adults with ARHL. A key strength of the study lies in the research team’s expertise in caring for older adults, as the team included nursing faculty and nurses with extensive clinical experience in gerontological care. This background enabled the interviewers to establish rapport with participants and to recognise the nuanced challenges embedded in older adults’ everyday lives, which contributed to the development of themes encompassing the psychosocial and functional aspects of ARHL during analysis. In turn, the authors’ experience supported the in-depth identification and interpretation of participants’ unmet hearing healthcare needs. The study also lays the groundwork for developing effective interventions to improve the quality of life of older adults with ARHL by providing evidence to inform hearing healthcare.

However, this study had several limitations. First, the participants were recruited through purposive sampling from a single senior welfare centre, which may have introduced selection bias. Oldre adults with ARHL in other community or healthcare contexts may have different experiences, thereby limiting the generalisability of the findings. Second, the participants were all Korean, potentially restricting cultural diversity. Future research should include participants from diverse settings, including rural and urban areas and varied ethnic groups. Third, we did not consider the severity of hearing loss. Therefore, differences in experiences according to hearing loss severity and between unilateral and bilateral hearing loss should be further explored. Finally, as a qualitative study based on in-depth interviews, the findings reflect participants’ subjective accounts and may be influenced by recall and self-report biases, which should be considered when interpreting the results.

## 9. Conclusions

This study used a phenomenological approach to explore the everyday communication experiences of older adults with ARHL. Our findings revealed specific difficulties and unmet needs, the process of accepting the chronic condition, and both positive and negative adaptations to hearing loss. Understanding the meaning of ARHL for aging populations provides a strong foundation for developing effective interventions that promote positive acceptance and coping strategies, ultimately enhancing their quality of life.

## Data Availability

The datasets analysed in the current study are not publicly available, but are available from the corresponding author on reasonable request.
